# COVID-19 Variants and Vaccine Development

**DOI:** 10.3390/v16050757

**Published:** 2024-05-10

**Authors:** Ziyao Zhao, Sahra Bashiri, Zyta M. Ziora, Istvan Toth, Mariusz Skwarczynski

**Affiliations:** 1 School of Chemistry and Molecular Biosciences, The University of Queensland, Brisbane, QLD 4072, Australia; ziyao.zhao@uq.net.au (Z.Z.); s.bashiri@uq.edu.au (S.B.); i.toth@uq.edu.au (I.T.); 2 Institute for Molecular Bioscience, The University of Queensland, Brisbane, QLD 4072, Australia; z.ziora@uq.edu.au; 3 School of Pharmacy, The University of Queensland, Woolloongabba, QLD 4102, Australia

**Keywords:** COVID-19, SARS-CoV-2, mutations, variants, vaccination, vaccine effectiveness

## Abstract

Coronavirus disease 2019 (COVID-19), the global pandemic caused by severe acute respiratory syndrome 2 virus (SARS-CoV-2) infection, has caused millions of infections and fatalities worldwide. Extensive SARS-CoV-2 research has been conducted to develop therapeutic drugs and prophylactic vaccines, and even though some drugs have been approved to treat SARS-CoV-2 infection, treatment efficacy remains limited. Therefore, preventive vaccination has been implemented on a global scale and represents the primary approach to combat the COVID-19 pandemic. Approved vaccines vary in composition, although vaccine design has been based on either the key viral structural (spike) protein or viral components carrying this protein. Therefore, mutations of the virus, particularly mutations in the S protein, severely compromise the effectiveness of current vaccines and the ability to control COVID-19 infection. This review begins by describing the SARS-CoV-2 viral composition, the mechanism of infection, the role of angiotensin-converting enzyme 2, the host defence responses against infection and the most common vaccine designs. Next, this review summarizes the common mutations of SARS-CoV-2 and how these mutations change viral properties, confer immune escape and influence vaccine efficacy. Finally, this review discusses global strategies that have been employed to mitigate the decreases in vaccine efficacy encountered against new variants.

## 1. Introduction

Coronavirus disease 2019 (COVID-19), caused by severe acute respiratory syndrome 2 virus (SARS-CoV-2), is an ongoing global pandemic that has threatened public health over the past 3 years. Quickly spreading throughout the world since 2019, the COVID-19 virus has infected over 760 million individuals and caused more than 6.9 million deaths worldwide. SARS-CoV-2 is a Beta-coronavirus from the family of *Coronaviridae* that contains a central core consisting of positive-sense, single-stranded RNA (+ssRNA) shielded by envelop proteins. Based on the serology studies and genomic analysis, the *Coronaviridae* family is classified into four genera: Alpha-, Beta-, Gamma- and Delta-coronaviruses, but only the Alpha- and Beta-coronaviruses are capable of infecting humans [1]. There are four common human coronaviruses (HCoVs): Alpha-coronaviruses HCoV-229E and HCoV-NL63, and Beta-coronaviruses HCoV-Oc43 and HCoV-HKU1. These HCoVs can cause mild to moderate illnesses, such as common colds [2].

Prior to the COVID-19 pandemic, there were two other pandemics caused by Beta-coronaviruses: severe acute respiratory syndrome (SARS) in 2003, caused by severe acute respiratory syndrome coronavirus (SARS-CoV), and Middle East respiratory syndrome (MERS) in 2012, caused by Middle East respiratory syndrome coronavirus MERS-CoV. All three viruses exhibit high genetic similarity with SARS-CoV-2 sharing 79% and 50% genetic similarity with SARS-CoV and MERS-CoV, respectively [3].

A variety of mild to severe symptoms have been associated with COVID-19. Typical mild symptoms of COVID-19 include flu-like fever, cough and muscle aches that often resolve without medical care, while severe COVID-19 symptoms include coma, difficulty breathing and severe chest pain that require intensive medical care. Asymptomatic infection and some rare symptoms have also been associated with COVID-19 [4]. Like influenza, the primary modes of airborne transmission for SARS-CoV-2 are contact and droplet transmission [5]; therefore, infection can spread through particles and droplets of respiratory fluids expelled when an infected person coughs, sneezes or talks. These infected respiratory fluids are inhaled by people in close contact (i.e., typically within 1 m) or fall into the mouth, nose, or eyes of close contacts causing infection [6].

Early in 2023, the World Health Organization (WHO) renewed recommendations for therapeutics in the Therapeutics and COVID-19 Living Guideline [7]. Among the three approved drugs for non-severe COVID-19, only Nirmatrelvir-ritonavir (Paxlovid) is recommended by the WHO. In addition, the WHO recommends corticosteroids, interleukin-6 inhibitors and baricitinib for severe and critical COVID-19. However, all of these recommended drugs have limited applicability and efficacy [8,9]. Vaccination against COVID-19 has been widely recommended as a preventive method and proven safe and effective. The primary aim of vaccination is to induce antibody production, although the importance of cellular immunity is often overlooked. Early in the pandemic, mRNA, DNA and whole-virus-based vaccines were approved by the WHO for emergency use and widely applied globally; however, there are now more than 300 vaccine candidates in different stages of pre-clinical or clinical trials.

During vaccine development, the whole or specific parts of a pathogen are selected as antigens and used to stimulate the production of antibodies that can identify and neutralize the virus once infection occurs [10]. The antigen-coding gene of the SARS-CoV-2 spike (S) protein was selected as the main component of the developed mRNA and DNA-based viral vector vaccines. The mutation sites on S proteins can prevent antigen recognition and evade the immune system, reducing the effectiveness of vaccines. Therefore, the continuous emergence of new variants with more mutation sites on the S protein poses a significant threat to vaccine effectiveness. The numbers of COVID-19 infections are still increasing as there are ongoing COVID-19 outbreaks in multiple countries, despite the majority of these populations being vaccinated. Thankfully, the current COVID-19 fatality rate is lower than it was during the early stages of the pandemic [11].

## 2. SARS-CoV-2 Composition

**Figure 1:**
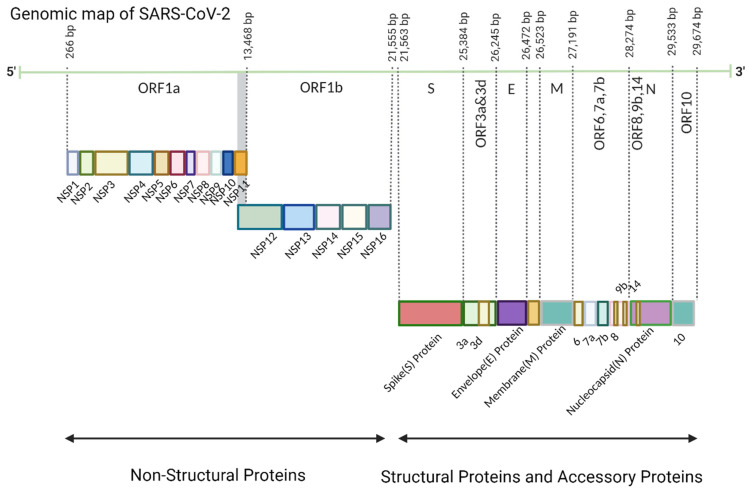
Genomic map of SARS-CoV-2. The number of base pairs indicates the position of various essential genomes that encode functional proteins.

The SARS-CoV-2 virus is composed of a core containing genetic material (i.e., positive-sense single-strand RNA (+ssRNA)) covered by a protein-based capsid. The genome of SARS-CoV-2 is approximately 30 kb with 10 functional open reading frames (ORFs) [12]. ORF1a and ORF1b occupy almost two-thirds of the whole genome sequence (about 20 kb) and are located near the 5′-cap end, while the other ORFs are close to 3′-poly(A) tail and code structural and accessory proteins [13]. All proteins encoded by the +ssRNA can be divided into three groups according to their functions: 16 non-structural proteins (NSPs), 4 structural proteins and 9 accessory proteins (Figure 1).

### 2.1. Structural Proteins

**Figure 2:**
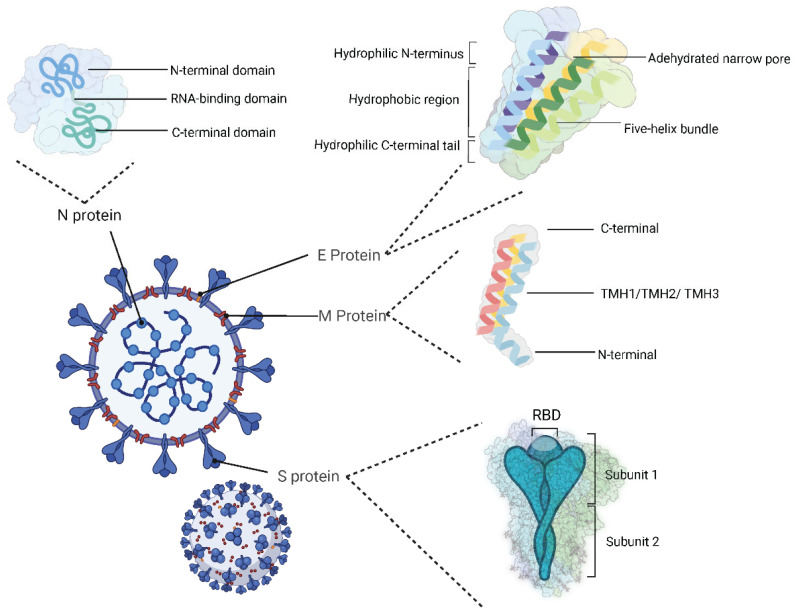
Structure of SARS-CoV-2.

The complete SARS-CoV-2 virion contains RNA and four structural proteins: spike (S), envelope (E), membrane (M) and nucleocapsid (N) proteins (Figure 2). Trimeric S protein (1273 amino acids) mainly consists of subunits 1 (S1) and 2 (S2) with the furin cleavage site located between the two subunits [14]. The whole protein structure begins with a signal peptide and ends with a transmembrane domain and cytoplasmic tail. Subunit 1, consisting of an N-terminal domain (NTD) and a receptor binding domain (RBD), mainly facilitates host cell recognition and receptor binding. Prior to viral entry into cells, the receptor binding motif (RBM) in the RBD interacts with angiotensin-converting enzyme 2 (ACE2) on the host cell surface [15]. The main function of S2 is membrane fusion facilitated by three functional domains that include the fusion peptide and heptad repeat 1 and 2 (HR1 and HR2) domains. The E protein has 75 amino acid residues, making it the smallest viral structural protein. Its overall structure consists of a five-helix bundle with a stick-shaped, dehydrated, narrow pore that contains a bipartite channel. Longitudinally, the structure is composed of a short hydrophilic N-terminal transmembrane (TM) domain linked with a large hydrophobic region, followed by a hydrophilic C-terminal domain. The protein functions include assembly and release of new virions, and the specific homopentameric structure plays major role in viral pathogenicity [16,17,18].

The most abundant and conserved structural protein in SARS-CoV-2 is the M protein. Composed of 222 amino acids, the M protein consists of a 19-amino-acid ecto-domain at the N-terminal domain, a long C-terminal endo-domain with 123 amino acid residues and three transmembrane helices (TMH1, TMH2, and TMH3) with 19, 25, and 25 amino acid residues, respectively [19]. With the help of the N protein, the M protein is primarily responsible for virus assembly and membrane budding [20,21,22,23,24]. In addition, interactions between M protein dimers induce conformational changes that influence virus assembly [24]. The N protein (419 amino acids) contains three conserved domains. The N- and C-terminal domains are predicted to be the β-strand, although the C-terminal domain also contains short helices. The link region between the two folded domains is known as the RNA-binding domain and is responsible for binding to the viral RNA genome [25,26]. In addition to being involved in viral RNA packaging as the structural protein, the N protein also promotes virion assembly and improves viral transcription efficiency.

### 2.2. Non-Structural Proteins

Non-structural SARS-CoV-2 proteins are produced following cleavage of the replicase polyprotein, which is generated from ORF 1a and ORF 1b. Some SARS-CoV-2 non-structural protein functions have been predicted based on the functions of analogous conserved proteins in SARS-CoV. In addition to functioning as enzymes, some non-structural SARS-CoV-2 proteins interact with other non-structural proteins and are involved in membrane remodelling, RNA replication and transcription (Table 1) [27,28,29,30,31,32,33,34,35,36,37,38,39].

### 2.3. Accessory Factors

Eleven SARS-CoV-2 proteins have been designated as accessory factors involved in a variety of functions, including inhibition of host immune responses, regulation of apoptosis and early evolution of the virus. Similar to predicting non-structural protein functions, the functions of accessory SARS-CoV-2 proteins have also been predicted based on their functions in SARS-CoV and other relevant coronaviruses, such as MERS-CoV. Compared with non-structural proteins, accessory factors are more variable between coronaviruses. The mutations of some accessory proteins have been associated with pathogenesis and increasing viral transmissibility [13,40].

## 3. Angiotensin-Converting Enzyme 2

Angiotensin-converting enzyme 2 (ACE2) is a receptor for the SARS-CoV-2 S protein and is widely expressed in multiple human tissues and organs, allowing the virus to enter and replicate in the lungs, intestines, kidney, gallbladder and heart [41]. The S protein has another target receptor on human host cell surfaces—the leucine-rich repeat containing 15 (LRRC15) receptor, which was identified as an inhibitory attachment factor for viral entry [42]. During infection, the S protein binds to ACE2 during the first stage of viral entry into the host cell [43]. Notably, the binding affinity of the SARS-CoV-2 S protein to ACE2 is 10 - 20 times stronger than the SARS-CoV S protein to ACE2 and is considered the key factor for increasing viral infectivity [44]. Prior to being discovered as a cellular receptor for the SARS coronavirus, ACE2 was identified as the primary enzyme in the renin–angiotensin system [45]—the complex regulatory system responsible for controlling the cardiovascular system and hydroelectrolyte balance [46]. SARS-CoV-2 infection disrupts the renin–angiotensin system by downregulating the expression of ACE2 [47], weakening anti-inflammatory protection and leading to multiple organ injuries [48,49,50,51].

## 4. Mechanism of SARS-CoV-2 Infection

**Figure 3:**
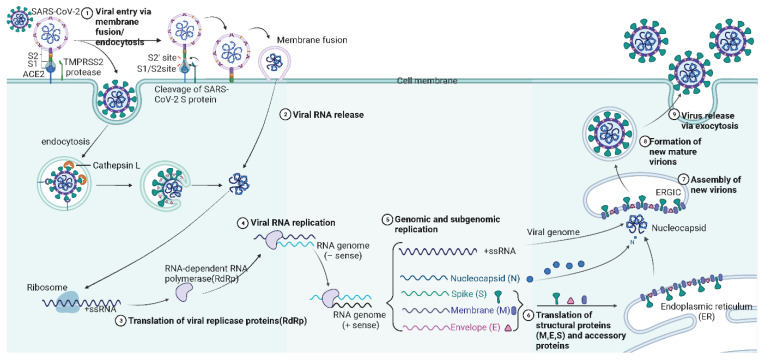
Mechanism of SARS-CoV-2 infection in the host cells. The SARS-CoV-2 S protein binds to the ACE2 receptor on the surface of host cells, the S1/S2 subunits are cleaved by the proprotein convertase furin. The S2’ cleavage site is exposed and further cleaved by the host cell protease transmembrane serine protease 2. Then, the viral cell entry via membrane fusion or endocytosis occurs, and RNA is released to the cytoplasm. Then, ribosomes from host cells are employed for the translation of RNA-dependent RNA polymerase before the viral RNA replication. RNA genome coding structural proteins are obtained. The structural proteins are translated into the host endoplasmic reticulum. Together with the new viral RNA and N proteins, the new virions are assembled in the ER-Golgi intermediate compartment (ERGIC) of host cells; finally, the new SARS-CoV-2 viruses are released outside the host cells through exocytosis.

Infection begins when the SARS-CoV-2 S protein binds to the ACE2 receptor on host cells and initiates viral entry. The mechanism of infection can be divided into nine steps (Figure 3) beginning with the virus binding to host cells and ending with the release of new viruses. Following recognition of the ACE2 receptor by the S protein, endocytosis and cell membrane fusion within the host cell are the next steps. The viral protein coat is then proteolyzed inducing the release of viral RNAs that serve as templates for RNA replication. The +ssRNAs promote translation of the viral replicase protein, known as RNA-dependent RNA polymerase (RdRp). The replicase proteins facilitate the synthesis of the negative strand by using the positive strand as a template. The synthesized negative strand enables replication of viral RNAs including both genomic and subgenomic RNAs. After the genomic and subgenomic replication, the host cell ribosome is used to express structural and accessory viral proteins—essential components of new viruses [52]. Structural viral proteins synthesized in the cytoplasm accumulate in the endoplasmic reticulum–Golgi compartment and are packed with replicated RNA to form a new virus. The newly formed virus is released by the cell through exocytosis to infect other surrounding host cells. The high demands of viral protein production cause the destruction of the host cell endoplasmic reticulum and eventually cell death [53,54,55]. The whole process is repeated in newly infected host cells, causing SARS-CoV-2 infection in the host and COVID-19.

## 5. Host Defence Mechanisms against Infections

The immune system is a complex network that helps defend the human body against pathogens. This defence system is based on innate and adaptive immunity. Non-pathogen-specific innate immunity is composed of physical and chemical barriers that act as the first line of defence to prevent pathogens from entering the human body. Physical barriers include skin, cilia, mucosa and their secretions; however, some pathogens evade these physical barriers and enter the human body, activating the second line of defence that involves the release of chemical defence signals and mobilisation of protective functional cells. Natural killer (NK) cells and phagocytic cells play major roles in chemical defence mechanisms. Complement proteins also participate in these defence systems. Unfortunately, non-pathogen-specific innate immunity is unable to prevent and resolve infection in many cases. In contrast, adaptive immunity is highly pathogen-specific and effective, although response time is longer compared to innate immunity. After pathogen recognition by the innate system, adaptive immune responses targeted to the specific pathogen antigens are triggered, initiating pathogen clearance and activating immunologic memory that can rapidly initiate strong humoral (antibody-based) and cellular (cytotoxic T lymphocyte-based) responses when the body encounters the same antigen again [56,57,58]. Adaptive immunity involves a complex network of immune cells and signalling molecules.

### Host Defence against SARS-CoV-2

**Figure 4:**
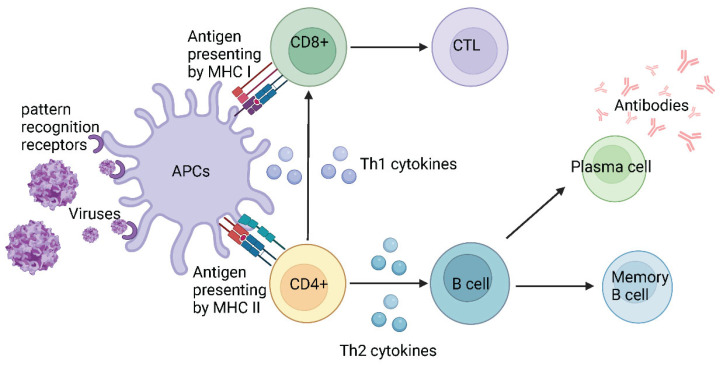
The simplified schematic representation of antiviral immunity.

Innate immunity plays an important role in controlling SARS-CoV-2 infection by limiting viral entry, translation, replication and assembly, facilitating recognition and clearance of infected cells and promoting adaptive immunity. Antigen-presenting cells (APCs), such as dendritic cells and macrophages, recognise the SARS-CoV-2 virus or pathogen-associated molecular patterns through pattern recognition receptors on the cell surface. Following recognition, antigens or pathogens enter APCs through endocytosis [59,60] and are presented to naive CD8+ T cells by corresponding major histocompatibility complex class I (MHC I) or to naive CD4+ T cells by MHC II [61]. There are two subsets of CD4+ T cells: T helper cells type 1 (Th1) and T helper cells type 2 (Th2), which release Th1 and Th2 cytokines, respectively. Th1 cytokines mainly stimulate CD8+ T cells to promote cellular immune responses and Th2 cytokines act on B cells to enhance humoral immune responses [62,63,64]. CD8+ cytotoxic T lymphocytes (CTLs) are responsible for killing and clearing infected cells, while B cells differentiate into memory B cells and plasma cells. Plasma cells produce antibodies that recognize specific antigens to prevent the infection and memory B cells can differentiate into plasma cells following future stimulation by the same antigens (Figure 4).

## 6. Vaccines

Vaccines play a crucial role in preventing infection and transmission of diseases. Vaccines typically consist of a pathogen or its fragments that stimulate adaptive immune responses, induce antibody production and activate cellular immunity, including cytotoxic T lymphocytes (CTLs).

### 6.1. Whole Pathogen Vaccines

Traditional whole pathogen vaccines contain inactivated/killed or attenuated/live pathogens. Attenuation greatly reduces pathogen virulency and its capacity to reproduce [65]. However, attenuated whole pathogen vaccines can produce infection under certain circumstances, such as in immunocompromised patients, exhibit some toxicity, and can induce undesired immune responses (e.g., excessive inflammation) [66]. Inactivated vaccines contain whole pathogens that have been killed and cannot replicate, and therefore do not continuously stimulate the immune system. While there is no risk of reversion to a virulent stage, inactivated whole pathogen vaccines are typically less immunogenic than attenuated [67]. Therefore, to achieve the desired immune response, killed pathogen vaccines often require more than one dose and/or the presence of an external adjuvant [68].

### 6.2. Subunit Vaccines

Subunit vaccines contain antigenic fragments of pathogens, such as proteins [69], peptides [64] or polysaccharides [70,71], that can elicit protective immune responses. Compared to traditional whole pathogen vaccines, subunit vaccines decrease the risks of side effects and are safer to use in immunosuppressed patients [72,73]. In addition, subunit vaccines do not require cultivation, making them more suitable for large-scale production than whole pathogen vaccines and reducing risks associated with biohazardous materials [72,73]. However, reducing the size of antigenic components can lead to decreased immunogenicity. Therefore, adjuvants and delivery systems are needed to enhance immune responses elicited by subunit vaccines [74]. As a result, a wide range of adjuvants and delivery systems have been developed, each with their own advantages and limitations, including (a) enhanced immunogenicity and longevity of responses vs. enhanced toxicity and ability to trigger excessive inflammatory responses [75], (b) ability to trigger humoral vs. cellular immunity, (c) cost and availability concerns, and (d) stability and storage conditions requirement [76,77,78].

### 6.3. Genetic Vaccines

Genetic vaccines are third-generation vaccines where the core antigenic components are genetic material (i.e., mRNA or DNA) [79] capable of expressing antigens. The genetic material is delivered to human cells that produce target proteins as antigens to stimulate an immune response [80]. The mRNA vaccine is a new type of genetic vaccine that has been developed in recent years and often uses both non-replicating (also referred to as conventional or non-amplifying) mRNA and self-amplifying RNA in the vaccine design. Non-replicating and self-amplifying RNA produce pathogen proteins using host cells. Compared with the traditional non-replicating mRNA vaccine, the self-amplifying mRNA vaccine contains additional viral replicase genes from alphaviruses or other virus species. The viral replicases promote rapid amplification of the whole mRNA, thus increasing the expression of protein antigens, allowing for the administration of vaccines at lower doses [81,82,83].

Initial development of mRNA vaccines was hindered by technical obstacles, including inherent structural instability of mRNA, low mRNA delivery efficiency and the potential of mRNA vaccines to induce severe inflammation in the human body. Several strategies have been employed to overcome these obstacles, such as genetically engineering mRNA to optimise the coding sequence, modifying purification methods to efficiently remove contaminating double-stranded RNA (dsRNA) and employing new materials that can efficiently deliver mRNAs and protect them from decomposition [84,85]. Modifying mRNA structure increases mRNA stability and translation efficiency while reducing immunogenicity [86]. In addition, lipidic, polymeric and peptide delivery systems can protect and deliver encapsulated mRNA into cells [87,88].

DNA vaccines contain DNA that encodes target proteins and delivery vectors that enable vaccine components to enter the cell nucleus where DNA is transcribed into mRNA for protein biosynthesis [79,89]. Compared with RNA vaccines that require specific cold storage conditions, DNA vaccines are more stable for transportation and can be stored at 2–8 degrees Celsius [90]. DNA vaccines can also induce both B and T cell immune responses; however, transportation of DNA to the nucleus presents a significantly greater challenge compared to delivery of RNA to the cytoplasm. To improve the effectiveness and safety of DNA vaccines, several improvements have included optimizing the structure of DNA constructs and delivery vectors [91]. Various DNA structures, including circular, linear and closed minimalistic forms, have been employed for vaccine development [92]. A typical circular DNA vaccine construct is plasmid DNA containing a gene encoding an antigen along with a promoter and terminator. Viral vectors have been widely used for genetic vaccine delivery and consist of a fragment of the target pathogen gene and a deactivated virus, unrelated to the targeted pathogen as a carrier [93]. These carriers have disadvantages, like toxicity and propensity to induce immunological reactions, that may lead to premature elimination of the vaccine before reaching its target. For example, an adenovirus is a typical vector with a highly immunogenic capsid that can trigger strong immune responses and severe inflammatory reactions [94], particularly following systemic administration of large doses [95,96]. However, the severity of these reactions can be reduced by genomic modification of the vector [95].

## 7. Vaccines against SARS-CoV-2

A wide variety of SARS-CoV-2 vaccines have been approved worldwide to either prevent COVID-19 infection or at least reduce disease severity. The WHO lists four types of COVID-19 vaccines approved for human use: mRNA vaccines, DNA-based viral vector vaccines, inactivated whole pathogen vaccines and protein subunit vaccines (Table 2).

### 7.1. mRNA Vaccines

The two most widely utilized mRNA vaccines are Pfizer-BioNTech (Comirnaty) and Moderna (Spikevax) COVID-19 vaccines. These non-replicating mRNA vaccines consist of nucleoside-modified mRNA encoding the full-length SARS-CoV-2 S protein packaged within synthetic, cationic lipid nanoparticles (containing polyethylene glycol (PEG) (Table S1) [97,98,99,100,101]. Notably, PEG-lipids enhance delivery efficacy and improve nanoparticle stability [98,102,103]. The vaccines are administered through a two-dose regimen and designed to prevent the SARS-CoV-2 S protein from binding to ACE2 receptors on host cells, blocking viral entry.

### 7.2. DNA-Based Viral Vector Vaccines

Three DNA-based viral vector vaccines have been approved by the WHO: Janssen COVID-19, CONVIDECIA COVID-19 and Vaxzevria. The Janssen COVID-19 vaccine is a single-dose vaccine that utilizes the recombinant non-replicating vector adenovirus type 26. CONVIDECIA COVID-19 is another single-dose vaccine that utilizes a modified, non-replicating adenovirus type 5 as its viral vector, while Vaxzevria consists of the non-replicating chimpanzee adenovirus ChAdOx and requires a booster dose. These viral vectors deliver genetic material encoding a stabilized version of the S protein to trigger an immune response against SARS-CoV-2.

### 7.3. Inactivated Whole Pathogen Vaccines

Four traditional inactivated whole pathogen vaccines have been approved by the WHO and are produced by purification of chemically inactivated SARS-CoV-2 cultured in a susceptible cell line [67,104]. However, the production of inactivated vaccines requires high biosafety level facilities, as cultivation of live infectious viruses and incomplete inactivation pose a risk of infection to personnel and the virus escaping from the facility [104]. These inactivated vaccines are usually adjuvanted with alum to improve immunogenicity (for example, in CoronaVac, BBIBP-CorV and VLA2001 vaccines) [105].

### 7.4. Protein Subunit Vaccines

Two protein-based subunit COVID-19 vaccines have been approved by the WHO: Nuvaxovid and Corbevax. Notably, Nuvaxovid is the first certified protein-based subunit vaccine that can confer protection against the different S proteins of SARS-CoV-2 variants. Novaxovid contains recombinant SARS-CoV-2 S protein nanoparticles, Matrix-M as the adjuvant and 40 nm cage-like nanoparticles composed of saponins, cholesterol and phospholipids [106].

Other types of vaccines are also being developed, like peptide-based subunit vaccines [107,108,109,110,111], live attenuated virus vaccines [112,113], bacterial vector vaccines [114], virus-like particle vaccines [115] and antigen-presenting cell-based vaccines [116,117].

## 8. Mutations and SARS-CoV-2 Variants

Viral mutations are found in a wide variety of viruses and are caused by substitutions, deletions or insertions of non-synonymous nucleotides in protein-coding genome sequences. Deleterious mutations do not strongly influence virulency as mutated viruses are often quickly eliminated. Neutral mutations are relatively safe for viruses and hosts as they do not change viral properties; however, certain mutations induce adaptive changes that increase viral infectivity and toxicity. Genetic mutations, and resulting variants, have been reported in SARS-CoV-2 since the beginning of the pandemic in 2020. Variants have been classified by the WHO into three categories: variant of interest (VOI), variant of concern (VOC) and variant under monitoring (VUM). Variant classifications differ between organizations, for example, the Centers for Disease Control and Prevention classify variants based on global impact.

### 8.1. Variants of Concern

Variants of concern (VOCs) exhibit increased transmissibility or virulence and negatively impact COVID-19 epidemiology and clinical presentation of disease, limiting the efficacy of public health measures or available diagnostics, vaccines and therapeutics (Table 3). The WHO has implemented various measures to address possible VOCs, including comparative assessments, laboratory investigations and sharing of virus isolates. For identified VOCs, primary actions include metadata sharing of complete genome sequences, field investigations and laboratory assessments. Since March 2023, Omicron parent lineages (i.e., BA.1, BA.2, BA.4/BA.5) and descendent lineages (XBB.1.5) were classified separately by the WHO as VOCs and VOIs to better identify different Omicron sub lineages.

### 8.2. Variants of Interest

Variants of interest (VOIs) contain mutations known or predicted to cause significant transmission in multiple countries and epidemiological impacts on global public health. To respond to potential VOIs, the WHO has described several primary actions including comparative assessment of variants, sharing virus isolates and information on relevant cases and monitoring and tracking the global spread. Previously circulating VOIs known to pose a reduced risk to global public health are listed and continuously monitored and evaluated. As of September 2023, circulating VOIs include EG.5, XBB.1.5 and XBB.1.16 from the XBB.1 sub-lineages with S protein mutations.

### 8.3. Variants under Monitoring

Variants under monitoring (VUMs) are SARS-CoV-2 variants with unclear phenotypic or epidemiological impacts that may be associated with future risks to global public health. All VUMs currently circulating are from the BA.2 sub-lineage with S protein mutations.

### 8.4. Variants and Mutation Trends

The number of mutation sites in SARS-CoV-2, particularly in RBD, is consistently increasing over time [118,119,120]. This can substantially alter viral features, while the increasing mutation frequency in the meantime causes a quickly growing number of non-lethal mutants circulating in the environment [120,121]. The main changes in viral phenotype are pathogenicity, infectivity, transmissibility and antigenicity [122]. Notably, mutations that increase viral infectivity exhibit greater dissemination and supplant less infectious viral phenotypes. Recombination has also been recently reported in newly emerging Omicron variants. Genetic recombination is the exchange of genetic material between objects that originate from distinct lineages. For example, XD—also designated as “Deltacron”—contains genomes from Delta (AY.4) and Omicron (BA.1) [123]. Recombination events can also occur within the same lineages, as exemplified by recombination of the Omicron XE variant through sibling lineages of Omicron (BA.1 and BA.2). In general, mutations are prevalent among viruses as they evolve and are subject to natural selection. During this progression, detrimental changes are eliminated and adaptive mutations are preserved. However, when mutations accumulate, variants deviate more noticeably from original and previously prevalent strains, creating unexpected challenges in the development of viral drugs and amplifying the complexity of infection management.

## 9. Impacts of Mutations

Mutations can alter the fitness and enhance the infectivity and pathogenicity of the SARS-CoV-2 virus. The SARS-CoV-2 S protein plays a crucial role in facilitating viral entry into the host cell, making the S protein a primary target of mutations that can directly impact viral characteristics. Several S protein mutation sites have been identified and found to be associated with the significant viral feature changes that increase the infectivity and transmissibility of SARS-CoV-2.

### 9.1. Mutations and Viral Characteristic Changes

The RBD, particularly the RBM, is the core part of the S protein that directly binds to ACE2 in host cells. Some mutations in the RBM can induce significant changes in SARS-CoV-2 phenotypes. For example, the S protein N439K mutation within the RBM was investigated early in the pandemic as this mutation enhances the binding affinity of the S protein to the ACE2 receptor and also reduces the neutralizing activity of some antibodies, including polyclonal and some neutralizing monoclonal antibodies, in sera from recovered patients. The diminished neutralization capacity of antibodies is considered an early manifestation of immune escape (described later in Section 9.2) [124,125]. Another mutation in the RBM, V483A, replaces a critical amino acid residue slightly changing the secondary S protein structure [126,127]. By changing the RBM of the S protein loop region, V483A indirectly affects receptor binding of SARS-CoV-2 [126] and increases the affinity of the S protein for ACE2, making this variant more infectious [128].

Some mutation sites outside of the RBD also change variant characteristics. For example, the D614G mutation replaces the 614th aspartic acid (S^D614^) of the S protein with glycine (S^G614^) [129,130,131], decreasing S1 release, increasing S protein incorporation into pseudovirions and increasing viral stability [132,133]. The D614G mutation also creates a new serine protease cleavage site in the S protein, significantly increasing the viral infectivity of ACE2-expressing cells [129]. In addition to viral transmissibility and infectivity being impacted by mutations, some mutations can also impact other properties of the virus. For example, the Y453F mutation not only enhances infectivity and transmissibility but is also resistant to convalescent sera, reducing the effectiveness of vaccines [134]. Mutations also occur in other structural proteins (Table 4), non-structural proteins and accessory factors. These mutations also alter viral properties with some associated with changes in variant nature. For example, I76F and L84S mutations in ORF8 directly impact protein–protein interactions with MHC-I [135] and disrupt antigen presentation and replication of the host interferon pathway, enhancing viral pathogenicity [135]. There are over 4000 mutations in the S protein [136]; however, only representative mutations are described in this review (and shown in Table 4). In general, mutations in viral proteins, particularly in the S protein, can strongly impact viral infectivity, virulence and immunogenicity and therefore require continuous monitoring.

### 9.2. Mutations Cause Immune Escape

The phenomenon known as immune escape, also referred to as immune evasion or antigenic escape, occurs when the host immune system fails to identify foreign antigens, preventing the immune system from initiating a response. This phenomenon directly correlates with pathogen (e.g., virus) evolution and survival. The RBM in the RBD is a key part of the S protein that directly binds to ACE2; therefore, more mutations in the RBM enhance the immune evasion properties of these variants (Table 4) [138,142,143,144,145,146,147,148]. For instance, it has been proposed that the D614G mutation can evade antibody neutralization [122,159]. Mutations in the E484 S protein residue can also evade antibody neutralization, including E484K mutations present in Beta and Gamma lineages [160,161,162], E484A mutations present in Omicron variants [163,164], and E484Q widely distributed in the Delta lineages [165]. However, the E484D mutation exhibits a relatively diminished capacity for immune evasion, showing resistance to only particular antibodies [166].

Mutations in proteins other than the S protein also cause immune evasion. Mutations in SARS-CoV-2 accessory proteins, such as mutations in ORF3 and ORF6, can perturb host innate immune responses and are therefore implicated in immune evasion [167]. Non-structural Nsp1 protein is considered a major immune evasion factor of SARS-CoV-2 as it blocks host antiviral defence proteins by interfering with cellular translation machinery [168]. Notably, a deletion in the coding region (Δ500–532) of Nsp1 reportedly triggers an even stronger immune escape of the variant [169].

### 9.3. Immune Escape Reduces Vaccine Effectiveness

**Figure 5:**
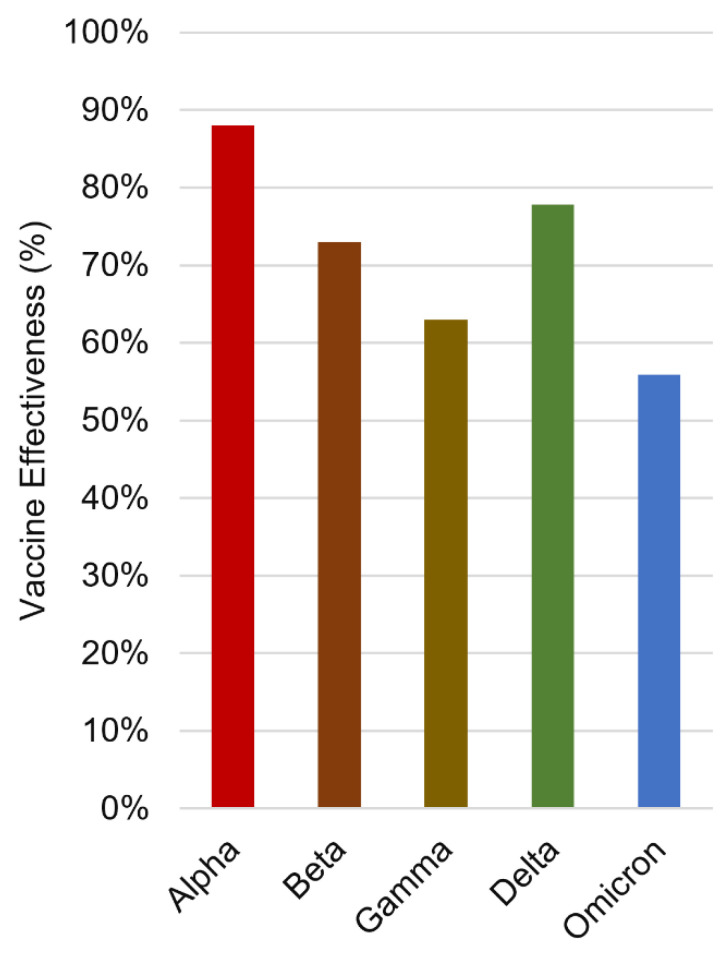
The impact of VOCs on the vaccine effectiveness. The summary of vaccine effectiveness is based on the Meta-analysis, the analysis (20 cohort studies with 52,782,321 participants, and 26 case–control studies with 2,584,732 cases) involves 11 vaccines (8 WHO-certified vaccines: Moderna, Pfizer, AstraZeneca, Johnson, Novavax, Sinopharm, Sinovac, Covaxin; 3 vaccines in development: SCB-2019, CVnCoV and HB02).

To date, vaccination has been the most effective medical intervention against COVID-19; however, mutations can impact antibody neutralization and reduce vaccine effectiveness [144]. A recent meta-analysis compared the impact of VOCs on the effectiveness of the most widely used vaccines targeted to the original SARS-CoV-2, including Pfizer, Moderna, AstraZeneca, Johnson, Sinovac, Sinopharm and Novavax (Figure 5) [170]. Decreased vaccine effectiveness weakens protection and creates greater public health challenges through the persistence and recurrence of COVID-19 outbreaks. To evaluate changes in vaccine effectiveness related to immune escape of variants, several comparative assessments of antibody neutralization in the sera from vaccinated individuals have been conducted [144].

#### 9.3.1. Impact of Immune Escape on Inactivated Vaccine Efficacy

The Sinovac COVID-19 vaccine is discussed here as a representative of inactivated vaccines. In a 2021 study, sera were collected 14 days after two doses of Sinovac and used to measure antibody-neutralizing activity against wild-type SARS-CoV-2 and variant forms. Compared with the antibody neutralizing activity against the wild-type virus, antibody neutralizing activity against Beta and Gamma variants was 5.3-, and 3.9-fold less, respectively [171]. Similarly, a more extensive study evaluated Sinovac vaccine effectiveness against VOCs and also showed reductions in sera antibody neutralizing activity by 2.9-, 5.5-, 4.3-, 3.4- and 12.5-fold against Alpha, Beta, Gamma, Delta and Omicron variants, respectively [172,173]. Another inactivated vaccine, the Sinopharm COVID-19 vaccine, has also exhibited reduced neutralization capacity against VOCs including Beta, Delta and Omicron variants with 6,1.9 and 11.2-fold reductions observed compared to the original virus [174].

#### 9.3.2. Impact of Immune Escape on Subunit Vaccine Efficacy

Novavax COVID-19 vaccine is the only protein-based subunit vaccine certified by the WHO. To evaluate the effectiveness of Novavax against immune-escape-related variants, sera were collected from individuals 14 days after vaccination with two doses of Novavax and used in antibody neutralization assays. Findings showed 8.1-, 41- and 30-fold reductions in neutralization capacity against Beta, Omicron BA.1, and Omicron BA.4/BA.5 variants compared to the strain with the D614G mutation [175].

#### 9.3.3. Impact of Immune Escape on Genetic Vaccine Efficacy

SARS-CoV-2 variants containing immune-escape-related mutations also reduce the effectiveness of genetic vaccines such as Moderna and Pfizer. The effectiveness of the Moderna vaccine against VOIs and VOCs, except Omicron, was evaluated in a test negative case–control study. The comparison was based on vaccine effectiveness against infection 14 days after the second dose. Results showed that effectiveness against infection with the Delta variant was 86.7% (95% confidence interval (CI): 84.3% to 88.7%), significantly lower than effectiveness against infection with the Alpha variant (98.4%, 95% CI: 96.9% to 99.1%) [176]. Compared with other variants, Delta variants with more immune-escape-related mutations further reduced vaccine effectiveness [176]. Another study also explored the Moderna vaccine’s effectiveness against Delta and Omicron variants 14–90 days after the second vaccination. Findings showed that vaccine effectiveness against infection with Omicron variants (44.0%, 95% CI: 35.1 to 51.6%) was lower than vaccine effectiveness against infection with Delta variants (80.2%, 95% CI: 68.2 to 87.7%) [177].

The effectiveness of the Pfizer COVID-19 vaccine, another widely used mRNA vaccine, has also been tested by determining the neutralizing titers against the original virus and VOCs (except the Omicron variant) in sera collected 14 days after the second dose. Compared to the original virus, results showed the following fold reductions in neutralizing titers for different variants: Alpha 1.7 (95% CI: 1.2–2.1), Gamma 2.3 (95% CI: 1.6–3), Beta 10.4 (95% CI: 6.4–14.4) and Delta 2.1 (95% CI: 1.7–2.5) [178]. The effectiveness of two doses of the vaccine in preventing hospitalizations for variants was 85% (95% CI: 82% to 88%) against the Alpha variant, 85% (95% CI: 83% to 87%) against the Delta variant and 65% (95% CI: 51% to 75%) against the Omicron variant [179].

**Figure 6:**
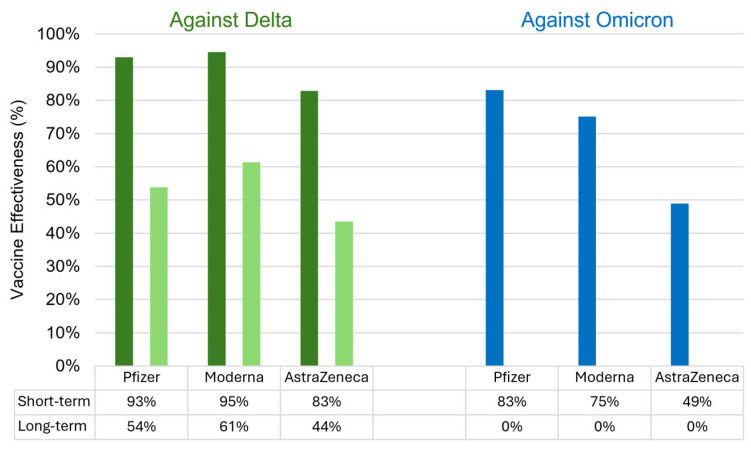
Pfizer, Moderna and AstraZeneca vaccine effectiveness against Delta (green bars) and Omicron (blue bars) variants. Short-term vaccine efficacy is marked in green/blue colour, while long-term efficacy in light green/blue colour.

Another problem associated with immune escape by variants is the decreased duration of protection provided by vaccines. A comprehensive literature review comparing the duration of effectiveness provided by Pfizer, Moderna and AstraZeneca demonstrated the impact of two variants with immune escape mutations, Delta and Omicron, on the quick decline in vaccine efficacy [180]. Vaccine efficacy was evaluated against Delta (B.1.617.2) and Omicron (B.1.1.529) variants in the short- (i.e., less 28 days after the second dose) and long-term (i.e., over 91 days after the second dose) and showed that vaccination protection over longer periods was significantly weaker (Figure 6).

### 9.4. Booster

Most marketed vaccines, except the Johnson single-dose vaccine, were originally designed to be two-dose regimes. However, due to declined efficacy against variants, especially Omicron, COVID-19 vaccine booster doses were recommended by the WHO and other government institutes. A booster is an extra dose administered after the vaccination regime (i.e., one or two doses) has been provided. Booster doses proved to be effective, at least to a certain extent, against Omicron variants [181]. There are two types of boosters: homologous and heterologous. Homologous boosters utilize the same vaccine administered as an additional dose, while heterologous boosters use a different vaccine for the additional immunization. Notably, as more sub-lineages of the Omicron variant emerge, updated bivalent vaccines are also being used as boosters.

#### 9.4.1. Homologous Booster

In a test-negative case–control study involving 2,663,549 participants, the effectiveness of the Pfizer booster against symptomatic disease caused by the Omicron (B.1.1.529) was explored. Vaccine effectiveness was 67.2% (95% CI: 66.5 to 67.8) at 2 to 4 weeks after the Pfizer booster, compared with primary vaccination effectiveness of 65.5% (95% CI: 63.9 to 67.0); therefore, the booster vaccination helped maintain short-term vaccine effectiveness similar to the primary vaccine. However, like the primary vaccination strategy, the effectiveness of the booster also decreased to 45.7% (95% CI: 44.7 to 46.7) over 10 weeks [182]. The Moderna mRNA vaccine booster efficacy was assessed by measuring neutralization antibody titers. Results showed that neutralization was enhanced by approximately 3.6-fold by a 100 μg booster after 2 weeks, compared to the neutralization titer measured 4 weeks after the second dose of the vaccine [183].

During the period of the pandemic when the Omicron variant predominated, the effectiveness of the Johnson vaccine against laboratory-confirmed COVID-19-associated emergency department or urgent care cases was 24% with one dose and 54% following administration of a homologous booster [184]. In the same study, vaccine effectiveness of single and booster strategies against laboratory-confirmed COVID-19-associated hospitalizations were 31% and 67%, again proving that homologous boosters provide higher protection than a single dose [184]. A regional study in Turkey among healthcare workers evaluated RBD immunoglobulin G (IgG) levels after homologous Sinovac booster administration and showed an 8.7-fold increase in antibody levels 4 months after the second dose [185]. Evidence shows that homologous booster administrations help confer protection against SARS-CoV-2, benefitting public health [186]. However, a persistent issue remains as the efficacy of vaccinations diminishes steadily over time.

#### 9.4.2. Heterologous Booster

Another strategy to reduce the effect of immune evasion involves heterologous boosters. After primary immunization with two doses of the Sinovac vaccine, homologous (i.e., Sinovac vaccine) and heterologous boosters (i.e., original monovalent Pfizer and AstraZeneca vaccines) were administered. The effectiveness of the homologous booster against hospitalisation and death was 59.3% (95% CI: 51.5–65.9%) and 62.7% (95% CI: 44.9–74.7%), respectively. However, heterologous boosters enhanced vaccine effectiveness more than homologous boosters with the Pfizer booster being 86.6% (95% CI: 83.6–89.1%) effective against hospitalisation and 90.7% (95% CI: 82.2–95.1%) effective against death. The heterologous AstraZeneca booster was slightly more effective than the Pfizer booster producing 93.0% (95% CI: 91.9–93.9%) and 94.7% (95% CI: 92.5–96.3%) effectiveness against hospitalisation and death, respectively [187]. A similar comparative study evaluated anti-RBD IgG levels after a two-dose Sinovac vaccination regimen with a homologous booster (i.e., Sinovac) and a heterologous booster (i.e., original monovalent Pfizer vaccine) and showed that anti-RBD IgG levels increased 8.7-fold and 104.8-fold, respectively [185].

Another study investigated booster effectiveness against Omicron variants in a population immunized with two primary doses of the AstraZeneca vaccine. The Effectiveness of the primary vaccines was lost 25 weeks following the second dose. A Pfizer booster restored protection to 62.4% (95% CI: 61.8–63.0%) while a Moderna booster restored protection to 70.1% (95% CI: 69.5–70.7%) 2–4 weeks following immunization. In addition, the effectiveness of the booster vaccines in the same population increased from 8.8% (95% CI, 7.0 to 10.5) over 25 weeks to 67.2% (95% CI: 66.5–67.8%) after the monovalent Pfizer booster and 73.9% (95% CI: 73.1–74.6%) after the monovalent Moderna booster (2–4 weeks following the booster) [182]. To determine the effectiveness of a heterologous fourth dose (i.e., one dose of the original monovalent Moderna vaccine after three doses of the original monovalent Pfizer vaccine), the level of Omicron-specific neutralizing antibodies was measured. Peak antibody titers after the fourth dose were similar to the peak antibody response following the third dose, suggesting that a heterologous fourth dose only restores immunity to peak levels rather than boosts immunity [188].

Numerous studies have demonstrated that heterologous boosting is more effective against variants than homologous vaccination [183,184,185,187,188]. The mechanism is not clear yet; however, may be attributed to the utilization of diverse antigens and adjuvants/delivery systems in primary and boosting vaccination, triggering various humoral and cellular immune responses [183], and cross-reactivity to variants preventing virus immunoescape [189,190].

#### 9.4.3. Bivalent Vaccines against Omicron Variants

The effectiveness of homologous and probable mechanisms of heterologous boosters has decreased as new Omicron sub-variants continue to emerge [191]. To mitigate the threat of new variants, new bivalent vaccines are developed. Compared to the original monovalent vaccines, bivalent vaccines are based on both the original SARS-CoV-2 strain and Omicron variants. Bivalent Omicron-containing boosters have been recommended as new heterologous boosters by multiple health institutions, including the European Medicines Agency, Therapeutic Goods Administration and the U.S. Food and Drug Administration. Initial bivalent vaccines were designed against BA.1 subvariant; however, as BA.4/BA.5 gradually replaced BA.1 and became the main circulating variants worldwide, the bivalent BA.4/5 vaccine was updated (Table S2) [192,193,194].

## 10. The Future of COVID-19 Vaccines

The major limitation of existing COVID-19 vaccines is poor efficacy against SARS-CoV-2 variants. Several approaches have been utilized to overcome this challenge including boosters, seasonal vaccination and novel vaccines utilising conserved antigen or antigens. The booster strategy for COVID-19 vaccination evolved from an initial monovalent immunisation to a bivalent approach where a different vaccine is used for the subsequent immunisation. However, boosters typically involved vaccines against old variants and only a small portion of produced antibodies could recognize new virus strains, making this strategy poorly and only temporarily effective.

An alternative approach involves cyclic production of new vaccines based on “updated” antigens that allow for continual viral mutation—an identical strategy to the current approach for controlling the spread of flu viruses. It is important to note that seasonal flu vaccines often exhibit moderate efficacy as their design is largely based on predicting what the predominant strain will be in the next flu season. Given the apparent seasonality of COVID-19 transmission, this approach is also the most feasible, albeit imperfect.

In theory, the most promising approach to overcome vaccination inefficacy against variants with immune escape mutations is substituting current non-conserved antigens with highly conserved alternatives. As previously stated, COVID-19 vaccine development mostly focuses on the SARS-CoV-2 S protein which exhibits a high propensity for mutation. However, other SARS-CoV-2 proteins may also be valid targets for vaccine development. The N protein sequence of SARS-CoV-2 has a 90.5% identity with SARS-CoV N protein, suggesting conservation [195]. Notably, the SARS-CoV N protein is also antigenic [196], making this protein a promising antigen for vaccine development [197]. Combining N and S proteins within a single vaccine has been suggested to reduce the impact of mutations on vaccine effectiveness [197]. With the help of immunoinformatic methods, a multi-epitope-based peptide vaccine was designed based on S and N proteins [198]. However, these vaccine candidates have not yet been tested in vitro or in vivo. Similarly, the bioinformatics analysis employed to design an N protein-based multi-epitope vaccine that can generate both humoral and cellular immune responses [199] has not been confirmed in vitro or in vivo. Furthermore, Crooke, S. N., et al. developed a computational workflow to identify and analyse the T and B cell epitopes derived from M, S and N proteins [200]. To date, this antigen replacement strategy has only been confirmed by bioinformatics analysis and still requires further experimental verification. Finally, it is worth noting that influenza A virus infection promotes SARS-CoV-2 infection and is associated with increased expression levels of ACE2 receptors [201]. This mechanism could also be explored to produce more effective COVID-19 vaccines [202].

## 11. Conclusions

The COVID-19 pandemic has caused a significant number of illnesses and fatalities on a global scale. Interactions between the spike protein of SARS-CoV-2 and ACE2 receptors are the fundamental basis for viral infection and vaccine development. Current vaccines confer some protection, although vaccine effectiveness against variants has decreased due to immune escape mutations. Among the variants, Omicron and its most recent variant, Kraken, exhibit the strongest capacity for immune escape, significantly reducing vaccine effectiveness. The efficacy of several techniques is currently being investigated to combat SARS-CoV-2, including booster strategies, the development of refreshed or seasonal COVID-19 vaccines and the substitution of antigenic components in vaccines with conserved antigens. However, all current strategies have limitations and are unable to overcome immune evasion exhibited by SARS-CoV-2 variants, so further innovative research is needed to mitigate these ongoing challenges.

## Figures and Tables

**Table 1:** Non-structural proteins of SARS-CoV-2.

Name	Range on Genome	Function	Sequence Reference Number
Nsp1	1–180	Leader protein host RNA inhibitor (degrade the host RNA) [27]	YP_009725297.1
Nsp2	181–818	Disruption of signalling in host cells (predicted based on SARS-CoV) [28]	YP_009725298.1
Nsp3	819–2763	Papain-like protease Promotion of RNA replication and transcription [29] Cleavage of proteins involved in the host innate immune immunity [30]	YP_009725299.1
Nsp4	2764–3263	Membrane remodelling (Binding to nsp3)	YP_009725300.1
Nsp5	3264–3569	3C-like proteinase Mediates cleavages downstream of nsp4 Cleavage of proteins related to the host innate immune response [30]	YP_009725301.1
Nsp6	3570–3859	Limitation of autophagic flux [31,32] Transmembrane domain	YP_009725302.1
Nsp7	3860–3942	Forms a complex with RdRp for genome replication [33]	YP_009725303.1
Nsp8	3943–4140	Combine with RdRp [33] Subunit of SARS-CoV-2 polymerase complex [34]	YP_009725304.1
Nsp9	4141–4253	RNA binding subunit [35]	YP_009725305.1
Nsp10	4254–4392	Regulator of viral replicase function Activation of methyltransferase activity as a co-factor [36]	YP_009725306.1
Nsp11	4393–4405	Unknown	YP_009725312.1
Nsp12	4393–5324	RNA-dependent RNA polymerase (RdRp) [37]	YP_009725307.1
Nsp13	5325–5925	Helicase [38]	YP_009725308.1
Nsp14	5926–6452	Exonuclease [38]	YP_009725309.1
Nsp15	6453–6798	Endoribonuclease [38]	YP_009725310.1
Nsp16	6799–7096	2′-O-methyltransferase Immune evasion related [39]	YP_009725311.1

**Table 2:** WHO-certified vaccines against COVID-19.

Vaccine	Developers and Country	Vaccine Type	Doses	Injection Interval *	Vaccination Restrictions **
Comirnaty	Pfizer-BioNTech (New York City, NY, USA and Mainz, Germany)	mRNA	2	21 days	All individuals aged 6 months and above
Spikevax	Moderna, NIAID (Cambridge, MA, USA)	mRNA	2	28 days	All individuals aged 6 months and above
Johnson & Johnson vaccine	Janssen Pharmaceuticals Johnson & Johnson (New Brunswick, NJ, USA)	Viral vector	1	-	18 and above
Vaxzevria	AstraZeneca, University of Oxford (Cambridge, UK)	Viral vector	2	28 days	18 and above
CONVIDECIA	CanSino Biologics Inc. (Tianjing, China)	Viral vector	1	-	18 and above
CoronaVac	Sinovac (Beijing, China)	Inactivated (Vero Cells)	2	14 days	18 and above; Further assessment for pregnant women is needed
BBIBP-CorV (Vero Cells)	Sinopharm (Beijing, China)	Inactivated (Vero Cells)	2	21 days	18 and above; Further assessment for pregnant women is needed
BBV152/COVAXIN	Bharat Biotech (Hyderabad, India)	Inactivated	2	28 days	18 and above; recommended for pregnant women only when the benefits of vaccination outweigh the potential risks
VLA2001	Valneva SE (Saint-Herblain, France)	Inactivated	2	28 days	18 to 50 years
Nuvaxovid	Novavax (Gaithersburg, MD, USA)	Protein Subunit vaccine	2	21 days	12 and above; recommended for pregnant women only when the benefits of vaccination outweigh the potential risks

* This interval was required for the vaccines that were originally designed as a primary booster model; ** Individuals with the allergic history of any component of the vaccine should not take the vaccines; Vaccination restrictions for pregnant women may be updated based on more data.

**Table 3:** VOCs circulating in 2020–2023.

WHO Label *	Pango Lineage **	GISAID Clade ***	Mutations in Binding Sites	First Reported (Date/Country)	Designation/Date
Alpha	B.1.1.7	GRY	S:N501Y	Sep/2020 UK	VOC: 18/Dec/2020 Previous VOC: 09/Mar/2022
Beta	B.1.351	GH/501Y.V2	S:K417N,S:E484K,S:N501Y	May/2020 South Africa	VOC: 18/Dec/2020 Previous VOC: 09/Mar/2022
Gamma	P.1	GH/501Y.V3	S:K417T,S:E484K,S:N501Y	Nov/2020 Brazil	VOC: 11/Jan/2021 Previous VOC: 09/Mar/2022
Delta	B.1.617.2	G/478K.V1	S:L452R,S:T478K,S:E484Q	Oct/2020 India	VOI: 4/Apr/2021 VOC: 11/May/2021 Previous VOC: 07/Jul/2022
Omicron	B.1.1.529	GR/484A	S:G339D,S:S371L,S:S373P,S:S375F,S:K417N, S:N440K,S:G446S,S:S477N,S:T478K,S:E484A, S:Q493R,S:G496S,S:Q498R,S:N501Y,S:Y505H	Nov/2021 Multiple countries	VUM: 24/Nov/2021 VOC: 26/Nov/2021 Previous VOC: 14/Mar/2023

* The Greek alphabet was recommended for non-scientific discussions by a WHO-convened expert group; ** Pango lineage is a classifying and naming system based on the virus genomes analysis, it is for genetically distinct lineages of SARS-CoV-2 and variants. Only the genomic sequence identified from the first cases is listed; the variety of lineage under the same label and the variations are not entirely consistent; *** GISAID is a nomenclature system for major clades to understand the patterns and determinants of SARS-CoV-2 variant strains, which are based on Pango lineage, the system contains 8 high-level phylogenetic groupings: the early split of S and L, the further evolution of L are V and G, the further evolution of G are GH, GR and GV, and the further evolution of GR are GRY.

**Table 4:** Representative mutations of S protein and impact on viral characteristics.

Mutations	Location	Impact
H49Y	NTD	Slight structural changes on the HR1 region; Increased cell entry [131]
V367F	RBD	Enhanced infectivity; Increased affinity to ACE2 [137]
K417N	RBD	Immune escape [138]
N440K	RBD (RBM)	Higher infectious fitness; Increased affinity to ACE2 [139,140]
S443A	RBD (RBM)	Stabilized SARS-CoV-2 S protein; Increased affinity to ACE2 [140,141]
K444R/Q/N *	RBD (RBM)	Immune escape [142]
L452R	RBD (RBM)	Immune escape [143,144]
Y453F	RBD (RBM)	Immune escape [145]
G476S	RBD (RBM)	Conformational changes in the S protein; Increased affinity to ACE2 [140]
T478K	RBD (RBM)	Immune escape [146]
S494P	RBD (RBM)	Immune escape [147]
N501Y	RBD (RBM)	Immune escape [148]
D839Y	S2 subunit	Structural changes on the S protein Enhanced infectivity (Prediction) [149]
D936Y	HR1	Conformational stability adjustment of the pre- and/or post-fusion S protein [150]
S943P	HR1	Conformational stability adjustment of the pre- and/or post-fusion S protein [150]
P1263L	Cytoplasmic tail	A significant increase in fusion (compared with WT) [151]
T9I	E protein	Destabilizing [152]
V62F	E protein	Highly destabilizing [152]
S68F	E protein	Slightly stabilizing [152,153,154]
R203K	N protein	Destabilized and decreased overall structural flexibility; Increased transmission and virulence of select SARS-CoV-2 variants [155,156]
G204R	N protein	Destabilized and decreased overall structural flexibility; Increased transmission and virulence of select SARS-CoV-2 variants [155,156]
D3G	M protein	A nonsynonymous substitution [157,158]
T175M	M protein	High antigenicity values [157,158]

* The substitutions are different in the sub-lineages of BA.2.

## Data Availability

No new data were created in this study. Data sharing is not applicable to this article.

## Supplementary Materials

The following supporting information can be downloaded at: https://www.mdpi.com/article/10.3390/v16050757/s1, Table S1: The ingredients of Comirnaty and Spikevax COVID-19 vaccine; Table S2. Bivalent vaccines. References [193,194,195] were cited in the Supplementary Materials.
