# Supplementary material for: COVID-19 Variants and Vaccine Development
Source: Viruses. 2024 May 10;16(5):757. doi: 10.3390/v16050757 (PMC11125726; doi:10.3390/v16050757)
Supplement: Supplementary file 1 [file viruses-16-00757-s001.zip › viruses-2999842-supplementary.pdf]

Table S1. The ingredients of Comirnaty and Spikevax COVID-19 vaccine.

Ingredients	Comirnaty (original)	Comirnaty (updated)	Spikevax
mRNA	Nucleoside-modified mRNA encoding the S glycoprotein of SARS-CoV-2		Nucleoside-modified mRNA encoding the S glycoprotein of SARS-CoV-2
Lipid	2[(polyethylene glycol (PEG))-2000]-N, N-ditetradecylacetamide<br>1,2-distearoyl-sn-glycero-3-phosphocholine<br>Cholesterol (plant derived)<br>((4-hydroxybutyl) azanediyl) bis(hexane-6,1-diyl) bis(2-hexyldecanoate)		PEG2000-DMG<br>1,2-distearoyl-sn-glycero-3-phosphocholine<br>BotaniChol®<br>SM-102
Other Ingredients	Dibasic sodium phosphate dihydrate<br>Monobasic potassium phosphate<br>Potassium chloride (common food salt)<br>Sodium chloride (basic table salt)<br>Sucrose (basic table sugar)	Sucrose (table sugar)<br>Tromethamine<br>Tromethamine hydrochloride	Sodium acetate<br>Sucrose (basic table sugar)<br>Tromethamine<br>Tromethamine hydrochloride<br>Acetic acid (the main ingredient in white household vinegar)

Table S2. Bivalent vaccines.

	Developers (Name)	Dose (mRNA) /Volume	Vaccine Composition	Effectiveness against Omicron subvariants
Pfizer and BioNTech (Comirnaty) Bivalent Booster	Original/Omicron BA.1 bivalent vaccine	30 µg/0.3 mL	15 µg WT+ 15 µg Omicron BA.1 (mRNA)	Higher neutralization activity against BA.2 and BA.5 compared with WT group<br>Decreased neutralization activity against BA.5 [193]
	Omicron BA.4/BA.5-adapted bivalent vaccine	30 µg/0.3 mL	15 µg WT+ 15 µg Omicron BA.4/BA.5 (mRNA)	Higher neutralizing responses against BA.5-derived sublineages (BA.4.6, BQ.1.1, and XBB.1) and BA.2-derived sublineage (BA.2.75.2) compared with the original monovalent vaccine [194]
Moderna (Spikevax) Bivalent Booster	BA.1 Omicron- containing vaccine (mRNA-1273.214)	50 µg/0.5mL	25 µg WT+ 25 µg Omicron BA.1 (mRNA)	Elicited neutralizing antibody responses against omicron that were superior to those with original monovalent vaccine [194]
	BA.4/BA.5 Omicron-containing vaccine (mRNA-1273.222)	50 µg/0.5mL	25 µg WT+ 25 µg Omicron BA.4/BA.5 (mRNA)	Enhanced neutralizing antibody responses against omicron sublineages (BA.1, BA.2.75.2 and BA.5) compared with original monovalent vaccine [195]

193. Kawasuji, H.; Morinaga, Y.; Tani, H.; Saga, Y.; Yamada, H.; Yoshida, Y.; Takegoshi, Y.; Kaneda, M.; Murai, Y.; Kimoto, K. Efficacy of the wild-type/Omicron BA. 1 bivalent vaccine as the second booster dose against Omicron BA. 2 and BA. 5. *medRxiv* **2022**, *preprint*

194. Zou, J.; Kurhade, C.; Patel, S.; Kitchin, N.; Tompkins, K.; Cutler, M.; Cooper, D.; Yang, Q.; Cai, H.; Muik, A. Improved neutralization of Omicron BA. 4/5, BA. 4.6, BA. 2.75. 2, BQ. 1.1, and XBB. 1 with bivalent BA. 4/5 vaccine. *BioRxiv* **2022**, *preprint*.
195. Scheaffer, S.M.; Lee, D.; Whitener, B.; Ying, B.; Wu, K.; Liang, C.-Y.; Jani, H.; Martin, P.; Amato, N.J.; Avena, L.E. Bivalent SARS-CoV-2 mRNA vaccines increase breadth of neutralization and protect against the BA. 5 Omicron variant in mice. *Nature medicine* **2023**, *29*, 247-257.
